# Edible Fungi and Skin Redox Homeostasis: Bioactive Diversity, Processing, Oral Exposure, and Evidence Gaps

**DOI:** 10.3390/antiox15091111

**Published:** 2026-09-02

**Authors:** Caizhen Wang, Ying Wang, Hongyu Chen, Bing Li, Youran Shao, Gen Zou

**Affiliations:** National Engineering Research Center of Edible Fungi, Key Laboratory of Applied Mycological Resources and Utilization, Ministry of Agriculture, Institute of Edible Fungi, Shanghai Academy of Agricultural Sciences, Shanghai 201403, China; 13524869286@163.com (C.W.); wyhrx@126.com (Y.W.); chenhongyu20128@163.com (H.C.); libing@saas.sh.cn (B.L.); yrshao@saas.sh.cn (Y.S.)

**Keywords:** ergothioneine, oxidative stress, redox homeostasis, Nrf2, photoaging, mushroom bioactives, food processing, oral bioavailability, gut–skin axis, dose realism

## Abstract

Oxidative stress drives skin aging, barrier impairment, and inflammatory amplification, making dietary antioxidants potential systemic contributors to cutaneous redox homeostasis. Edible fungi are distinctive sources of redox-active metabolites, particularly ergothioneine, a stable sulfur-containing antioxidant whose cellular uptake is mediated by the ergothioneine transporter OCTN1 (SLC22A4). This review evaluates ergothioneine, polysaccharides and β-glucans, cordycepin, phenolics, and *Ganoderma* triterpenoids as processing-sensitive dietary bioactives with redox relevance rather than topical cosmetic ingredients. We examine how cultivation, drying, cooking, extraction, fermentation, and microbial biomanufacturing determine antioxidant formation, retention, oral bioaccessibility, and dose realism, and how antioxidant response, inflammatory, mitochondrial, and gut microbiota-mediated pathways connect intake to skin endpoints. The strongest oral evidence concerns biomarker-linked ergothioneine-rich *Pleurotus*. Smaller *Flammulina velutipes* and *Sparassis crispa* trials report hydration or transepidermal water loss signals without comparable exposure biomarkers, whereas purified ergothioneine provides provisional non-mushroom food evidence. Compared with better-established oral ingredients, edible fungi offer distinctive food technology advantages but a narrower human evidence base. Priorities include processing-aware quality and safety markers, contaminant control, standardized digestion models, dose-realistic exposure estimates, and biomarker-anchored randomized human trials.

## 1. Introduction

Skin health and appearance are increasingly understood as outcomes not only of topical care but also of nutrition, systemic redox balance, immune status, and gut–skin interactions [1,2], which has driven rapid growth in oral, food-based approaches to skin-targeted nutrition [3]. Within this landscape, edible fungi occupy a distinctive but under-examined position: they are widely consumed foods with a long cultural association with vitality and healthy aging, and they are unusually rich in bioactives, including ergothioneine, polysaccharides and β-glucans, cordycepin, phenolics, and triterpenoids, that are mechanistically relevant to skin biology [4]. Yet most of the existing literature treats mushrooms either as topical cosmetic ingredients or as sources of isolated compounds, leaving their behavior as oral, processing-sensitive food systems largely unexplored [5,6]. From a food science perspective, the key questions are not only whether fungal bioactives are biologically active, but whether they are formed, preserved, released, absorbed, and delivered to skin-relevant targets after realistic dietary intake, and whether they can be standardized and produced safely at food scale. Accordingly, this review follows the route from fungal bioactive formation and processing through oral exposure to skin-relevant evidence, safety, and translation.

### 1.1. Wood-Decay Redox Ecology, Its Limits, and Traditional Food Narratives

The scientific story of edible fungi and skin-targeted nutrition can begin before food, cosmetics, or consumer trends: a substantial subset of edible or food-compatible basidiomycetes evolved in lignocellulose-rich habitats. Wood is protected by lignin, an aromatic polymer that makes plant cell walls resistant to microbial attack. Comparative genomics indicates that enzymatic lignin decomposition emerged early in Agaricomycetes and that lignin-degrading peroxidases expanded in lineages leading to white-rot fungi [7]. Many familiar food fungi, including *Pleurotus* and *Lentinula*, were therefore shaped by the biochemical challenge of accessing carbon locked within lignified plant cell walls.

Accessing wood polymers frequently requires extracellular oxidative chemistry. Because many hydrolytic and oxidative enzymes are too large to penetrate intact lignified secondary cell walls, reactive oxygen species (ROS), especially hydroxyl radicals generated through Fenton-type reactions, can help initiate wood decay [8]. This strategy creates an apparent paradox: the same nonspecific oxidants that loosen plant cell walls can also damage fungal hyphae and secreted enzymes. Brown-rot studies show that wood-decay fungi limit this risk by spatially and temporally controlling Fenton reactants, quenching ROS through antioxidant systems and secreting ROS-tolerant enzymes [9]. This provides a mechanistic ecological rationale for discussing wood-associated edible fungi through a redox biology lens.

High ergothioneine and glutathione contents have been reported in several edible basidiomycetes, including wood-decay or lignocellulose-associated taxa such as *Pleurotus*, *Lentinula,* and *Grifola* [4]. A useful boundary case is the ectomycorrhizal *Boletus edulis*, which can retain very high ergothioneine despite not executing autonomous wood decay. This case shows that redox-intensive substrate ecologies are an organizing lens for edible fungal chemistry, but not a deterministic explanation for ergothioneine distribution. Edible fungi further removed from autonomous lignocellulose oxidation, including *Agaricus* on composted substrate, *Cordyceps militaris* on insect hosts, and *Tremella fuciformis*, whose wood substrate is degraded by its companion ascomycete *Annulohypoxylon stygium*, generally sit lower [10,11,12,13]. We refer to this as a bounded redox division-of-labor framework, in which oxidative substrate attack, indirect access to wood-derived carbon and alternative chemistries such as polysaccharide accumulation are read as species-aware tendencies that admit clear exceptions.

With these limits made explicit, the ecological background provides context for traditional and contemporary wellness narratives around edible fungi. Such narratives do not substitute for controlled evidence; the relevant question is which fungal bioactives are formed, preserved, released, absorbed and ultimately linked to measurable skin outcomes after oral intake.

### 1.2. Why Another Review Is Needed

Four gaps motivate this review. First, the edible fungi/skin literature is still dominated by topical, cosmetic, pharmacological, and extract-based perspectives, in which mushrooms are treated mainly as ingredients applied to the skin surface: creams, films, topical antioxidant formulations, and enzyme-inhibition assays. Their behavior as foods that are consumed and act systemically has received far less attention. Recent comprehensive reviews of macrofungal extracts in anti-aging skin care [5] and of mushrooms as cosmeceutical ingredients targeting elastase, tyrosinase, hyaluronidase, and collagenase [6] reinforce this orientation: both surveys evaluate *Ganoderma lucidum*, *Lentinula edodes*, *Pleurotus ostreatus*, *Agaricus bisporus*, *Tremella fuciformis,* and related species almost exclusively through topical or formulation endpoints, leaving the dietary route, food processing and oral bioavailability dimensions largely untreated.

Second, recent reviews either address oral skin health ingredients broadly or discuss mushrooms within the wider context of healthy aging. Ren, Liu, and Xu [3] reviewed orally administered nutricosmetics across diverse food sources, whereas Akinyede et al. [14] surveyed the nutritional and therapeutic potential of mushrooms in healthy aging and longevity, with emphasis on antioxidant and cytoprotective mechanisms, hallmarks of aging, and L-ergothioneine. Neither review specifically follows the skin-focused translational chain from fungal processing and oral bioaccessibility to dose realism, exposure biomarkers, human skin endpoints, and food safety standardization.

Third, the fungal food technology dimension remains under-developed. Unlike purified actives, mushroom-derived food materials are strongly shaped by species, strain, fruiting body versus mycelium, substrate, drying, cooking, extraction, fermentation, storage, and microbial biomanufacturing. These variables determine the concentration, stability, and standardization of fungal bioactives in real food systems, yet they are seldom integrated with skin-relevant mechanisms or clinical endpoints.

Fourth, a comparative context is needed. The oral skin health field already has better-developed evidence streams for collagen peptides, ceramides, carotenoids, polyphenols, and probiotic/prebiotic strategies. These ingredient classes help define what a mature evidence base looks like: defined composition, realistic intake levels, randomized human trials, and validated endpoints such as transepidermal water loss, stratum corneum hydration, elasticity, minimal erythema dose, and wrinkle parameters [15,16,17,18,19].

Selected comparator studies make the gap concrete. Oral hyaluronan trials have measured hydration, wrinkles, tone, or epidermal structure [20,21], lycopene/lutein supplementation has been evaluated with UV-responsive molecular endpoints [22], and *Lactobacillus paracasei* NCC 2461 has been tested for skin reactivity and barrier recovery [23]. These examples do not shift the focus away from edible fungi; they clarify the ingredient characterization, dose control, exposure biomarkers, and skin endpoints that mushroom-based nutrition needs.

### 1.3. Aim and Scope

This review critically evaluates edible fungi as food systems and biotechnology platforms for skin-targeted dietary nutrition. We emphasize: (i) the composition and formation of skin-relevant fungal bioactives; (ii) how processing, fermentation, and biomanufacturing shape their availability and standardization; (iii) oral bioaccessibility, bioavailability, and metabolism; (iv) plausible gut-to-skin mechanisms; and (v) translational challenges separating a biologically active extract from an evidence-based food ingredient. Topical applications are discussed as a complementary food–cosmetics interface, and topical findings are not used to support oral claims.

Throughout the review, we apply a route discipline principle: mechanistic plausibility in cell or animal systems is not equivalent to oral efficacy in humans; topical evidence cannot be extrapolated to dietary intake without digestion, bioaccessibility, absorption, systemic distribution, or gut-mediated signaling; and oral skin benefit claims require route-relevant exposure evidence, ideally including biomarkers that connect intake to systemic or tissue-level readouts. Later sections refer back to this principle rather than restating the full logic in each bioactive class.

**Figure 1:**
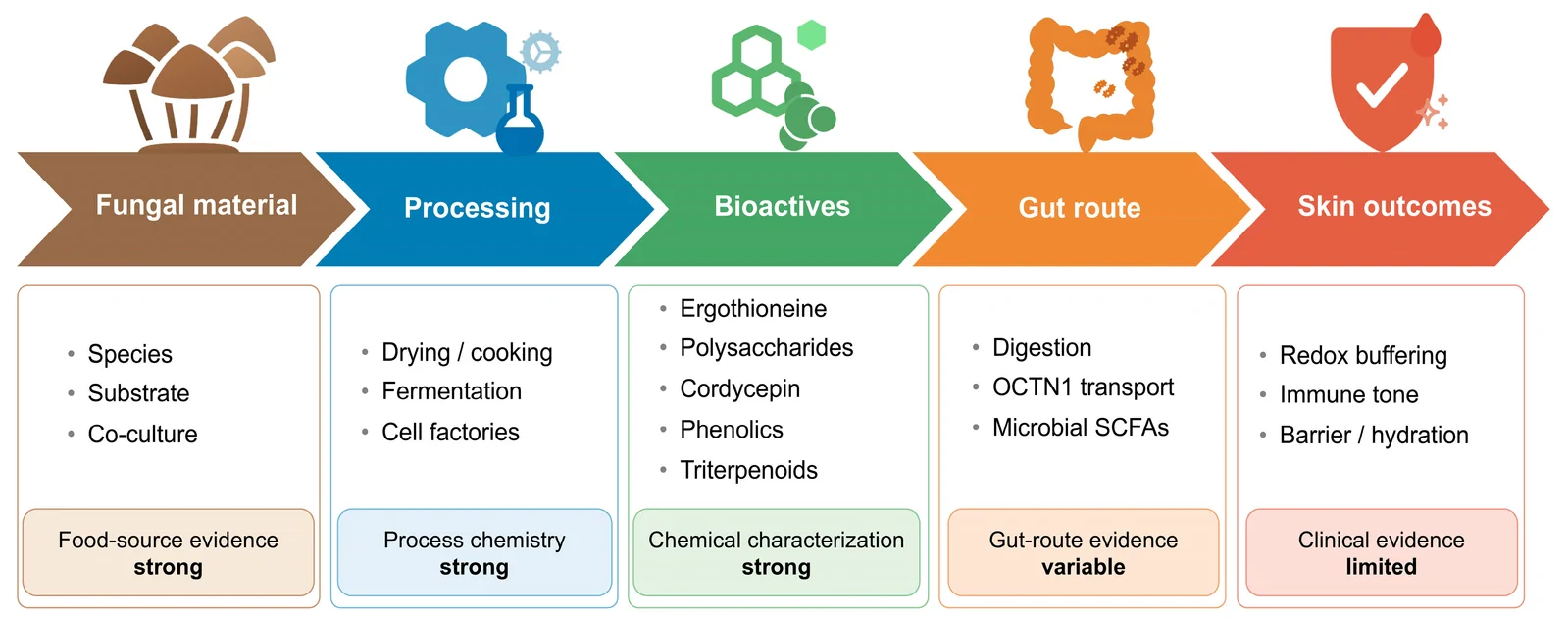
Integrated route map linking edible fungi to skin-targeted dietary nutrition. Fungal species, strain, substrate, and co-culture context define the raw material; processing and production shape the bioactive profile; digestion and gut microbial transformation determine oral exposure; and systemic delivery or microbial/immune mediators connect intake to skin endpoints.

The present scarcity of dietary human evidence provides the organizing rationale for the review. By appraising what is and is not known for each bioactive class, making the evidence gaps explicit and translating them into a processing-aware, route-validated research framework, we aim to define a testable food science framework for edible fungi. Figure 1 summarizes this route from fungal raw material to skin endpoints and frames the structure of the review.

### 1.4. Literature Search Strategy and Study Selection

The literature base was assembled through a structured identification and selection process rather than a formal systematic review or meta-analysis following PRISMA reporting standards [24]. Web of Science Core Collection, Scopus, and PubMed were searched for records published from January 2000 to 29 June 2026, supplemented by backward citation searching and targeted searches for processing, dose realism, oral exposure, safety, and route-relevant skin outcomes. Additional contextual sources were included when they directly informed edible fungi functionality, food-derived bioactives, fermentation, gut barrier mechanisms, medicine–food homology, or translational standardization. Details of the search logic and screening criteria are provided in Supplementary Table S1. During revision, one review published on 29 June 2026 and identified during peer review was added to contextualize the novelty of the present synthesis. The final reference set therefore comprised 106 references.

Records were included when they concerned edible or food-compatible fungi, or fungal origin bioactives belonging to the classes considered here, and addressed at least one analytical dimension of the review: bioactive formation and composition, food processing, fermentation or biomanufacturing, oral bioaccessibility or bioavailability, gut-to-skin mechanisms, skin-related endpoints, dose realism, standardization, or food safety. Human intervention studies, animal oral studies, in vitro digestion and microbiota models, mechanistic studies relevant to oral-route interpretation, food technology and analytical studies, and selected comparator evidence from better-established oral skin health ingredients were retained. Purely topical or cosmeceutical studies without oral or mechanistic relevance, non-food-relevant fungi, unretrievable full texts, and off-topic records were excluded or used only as contextual background.

### 1.5. Evidence-Grading Framework

To give readers a consistent evaluative lens before the compound-specific sections, this review applies a five-level evidence hierarchy that ranks studies by route, material definition, endpoint strength, and exposure verification. The framework operationalizes the route discipline principle defined in Section 1.3, is summarized in Table 1, and is applied throughout the subsequent tables and in the discussion of oral, animal, mechanistic, and topical evidence.

## 2. Edible Fungi as Processing-Sensitive Food Systems

### 2.1. Why Edible Fungi Differ from Plant-Derived Nutricosmetics

Edible fungi occupy an unusual position among food-derived sources of skin-relevant bioactives. A wood-associated subset, particularly several edible basidiomycetes, has life histories involving oxidative substrate attack and oxidative damage control, which supports a redox biology lens for those species without extending the same ecology to all edible fungi. Within the same raw materials, high-molecular-weight polysaccharides coexist with defined low-molecular-weight compounds such as ergothioneine and cordycepin. Their long dietary history sits alongside documented bioactive diversity, and their composition is unusually sensitive to cultivation and processing. Production can also shift among fruiting bodies, mycelia, liquid fermentation, and engineered microbial systems, while stems, residues, spent substrates, and side-streams create opportunities for circular food ingredient valorization.

The practical consequence is that edible fungi behave as variable food materials whose composition shifts with how they are grown, handled, and processed. We propose framing them as modular bioactive systems whose composition is defined by species, strain, tissue, substrate, processing route, and extraction fraction. These systems can then serve as the raw material for processing-aware edible fungi ingredients: skin-targeted food materials whose potential claims are tied to a declared processing trajectory.

### 2.2. Major Fungal Resources and Bioactive Logic

A small set of edible or food-compatible fungi recurs across the food-and-skin literature, but these species should not be forced into a single ecology or evidence model. They are used here to anchor a process- and evidence-centered analysis, while the detailed species–bioactive relationships are summarized in Table 2.

*Tremella fuciformis* is a principal example in discussions of acidic heteropolysaccharides, hydration narratives, and gut–skin questions, but it sits outside the classic autonomous wood-decay model because commercial cultivation depends on the companion ascomycete *Annulohypoxylon stygium* to supply assimilable carbon [13]. *Pleurotus* spp. and *Lentinula edodes* represent wood-associated basidiomycetes with stronger ergothioneine and beta-glucan relevance, whereas *Cordyceps militaris*, *Ganoderma lucidum,* and *Auricularia* spp. anchor nucleoside, triterpenoid/polysaccharide, and dietary-fiber-like materials, respectively.

Regional food cultures partly explain the evidence skew: *Tremella* and *Auricularia* in East Asian cuisines, *Lentinula* in Japanese and Korean traditions, *Pleurotus* in European cultivation systems, *Cordyceps* in traditional Chinese medicine and functional food markets, and *Agaricus* in Western mass-market cultivation. Table 2 summarizes the five bioactive classes considered in this review, their food technology variables, plausible oral routes, skin-relevant targets, and evidence levels, as defined in Section 1.5.

## 3. Bioactive Classes and Skin-Relevant Targets

### 3.1. Ergothioneine: A Fungal Dietary Antioxidant with a Clear Chemical Identity

Among fungal small molecules, ergothioneine is a sulfur-containing histidine derivative carrying an imidazole ring, a sulfur group, and a methylated quaternary nitrogen. In contrast to compositionally heterogeneous macromolecules, it is a structurally defined single compound, which makes it well suited to quantification, quality control, and ingredient standardization.

**Scheme 1:**
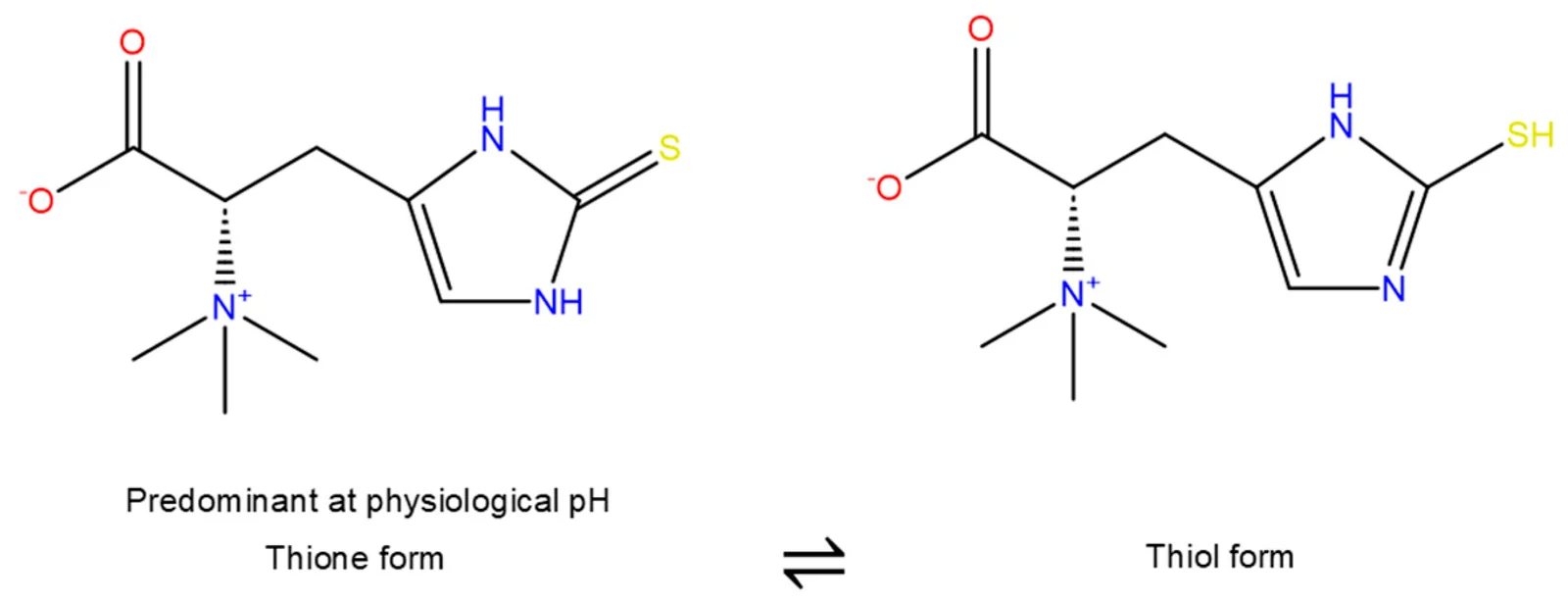
Thione–thiol tautomerism of L-ergothioneine. O, N, and S atoms are shown in red, blue, and yellow, respectively; the carbon framework is shown in black.

Functionally, its most important property is that, under physiological conditions, it exists predominantly as the relatively stable thione tautomer and not as the more readily oxidized thiol form. This thione–thiol tautomerism confers high stability and resistance to autoxidation relative to ordinary low-molecular-weight thiols [41], as illustrated in Scheme 1.

Ergothioneine is produced mainly by certain fungi and microorganisms, and edible mushrooms are among its most important dietary sources. Reported contents differ markedly among species, and distribution within a fruiting body is uneven, with caps generally exceeding stems. The dietary relevance of ergothioneine for skin is reinforced by its transport biology: dedicated transport via OCTN1/SLC22A4 allows accumulation in skin-relevant cell types, and the transporter is considered both a controller and an indicator of ergothioneine activity [27,42,43], positioning it as a systemically delivered, skin-accessible antioxidant. On this basis, ergothioneine has been argued to function as an under-recognized dietary micronutrient relevant to healthy aging [44]. A recent mushroom-focused healthy aging review likewise highlights ergothioneine as a major antioxidant and cytoprotective constituent [14]. Crucially, ergothioneine is a broad fungal and microbial cytoprotective compound; its abundance in mushroom foods is what makes it especially relevant to skin-targeted nutrition.

The food-source argument is also stronger for ergothioneine than for most other fungal skin-relevant compounds. Cultivated and wild mushrooms differ markedly in ergothioneine and glutathione content, with reported ergothioneine values spanning roughly 0.4–2.7 mg/g dry weight in *Lentinula edodes* and *Pleurotus ostreatus*, up to about 7 mg/g in *Boletus edulis*, but markedly lower values in *Agaricus bisporus* and only tens of micrograms per gram in *Tremella fuciformis* [4,10,35]. Analytical studies have repeatedly shown that species such as *Pleurotus*, *Agaricus,* and *Lentinula* can contribute meaningful dietary ergothioneine relative to ordinary background intake [4,35]. This does not imply that usual mushroom consumption reaches trial-level doses, and the high *Boletus* value reinforces that ergothioneine distribution cannot be reduced to wood decay alone; rather, it allows dose realism to be quantified instead of assumed (Section 5.4).

The food health relevance of ergothioneine is further supported by evidence that ergothioneine treatment improved intestinal barrier integrity and activated intestinal autophagy in *Drosophila*, extending the compound from a redox-active dietary metabolite to a gut barrier and healthy-aging candidate [45]. For skin-targeted dietary nutrition, this finding does not establish human skin efficacy, but it strengthens the logic for measuring gut barrier and exposure biomarkers alongside skin endpoints.

A further reason ergothioneine is unusually well positioned for skin-targeted nutrition is its accumulation in, and potential protection of, mitochondria. Mitochondrial dysfunction is a recognized contributor to skin aging through declining ATP supply, increased mitochondrial ROS leakage, and impaired keratinocyte and fibroblast renewal. Mass spectrometric work has demonstrated that ergothioneine can be taken up into isolated mitochondria from cells and tissues, and that OCTN1-independent transport routes may contribute to this uptake, supporting a mitochondrial-protective action that extends beyond cytosolic antioxidant chemistry [46]. Mechanistic and aging-focused reviews further link ergothioneine to mitochondrial integrity, Nrf2-mediated antioxidant gene programs, and longevity-associated pathways [47]. For dietary skin nutrition, this suggests a plausible food-to-organelle chain: a fungal dietary metabolite is absorbed, transported into skin-relevant cells, and could attenuate mitochondrial oxidative stress, a process relevant to cutaneous aging.

### 3.2. Polysaccharides and β-Glucans: Defined but Variable Dietary Macromolecules

Polysaccharides are the most extensively studied fungal macromolecules, occurring in fruiting bodies, mycelia, and fermentation broths/exopolysaccharides. They form a family of homo- and heteropolysaccharides. The most representative structural unit is the cell wall β-glucan, typically a β-1,3-linked backbone with variable β-1,6 branching, accompanied by mannogalactans, xylomannans, and glucuronic-acid-containing acidic heteropolysaccharides.

Across representative species, the structural diversity is large [30]. Shiitake lentinan is a neutral β-1,3-glucan with β-1,6 branches and higher-order conformations. *Tremella* polysaccharide is generally classed as an acidic heteropolysaccharide dominated by mannose, xylose, and glucuronic acid, with charge and rheological behavior distinct from β-glucans [13]. *Auricularia* polysaccharides are more heterogeneous, with monosaccharide ratios varying with origin, processing, and extraction. *Pleurotus* materials can yield glucans and branched mannogalactans, and sequential cold-water/hot-water/alkali extraction produces distinct fractions [30].

This need for structural discipline is reinforced by recent medicine–food homology reviews of *Poria cocos* and *Ganoderma* polysaccharides, which emphasize structural characterization, molecular weight distribution, monosaccharide composition, and bioactivity relationships rather than total polysaccharide content alone [48]. More broadly, polysaccharide-focused food health reviews, including those on edible fungus hypoglycemic activity and plant-derived polysaccharide mechanisms, show why macromolecular bioactives require mechanism-aware interpretation before being translated into functional food claims [49,50].

For oral use, fermentability and immune interaction are the decisive properties, and topical film formation is largely beside the point. Molecular weight, branching, charge, solubility, and purity determine how fungal polysaccharides are released from food matrices, transformed by gut microbiota and connected to short-chain fatty acid production, inflammatory tone, and barrier-related outcomes.

Recent discussion of edible fungi polysaccharides has further focused on intestinal mucus as a key entry point for functional activity [51]. This perspective is useful for skin-relevant nutrition because mucus integrity, epithelial immune sensing, and microbial access may determine whether fungal polysaccharides remain local gut substrates or generate systemic barrier-relevant signals. Consistent with this view, food polysaccharide studies have linked gut microbiota, mycobiome, metabolome, and epithelial regeneration to systemic or barrier-related outcomes [52,53].

β-Glucan biology adds a mechanistic anchor. Dectin-1 was first identified as a receptor for β-glucan [32], and fungal β-glucans are now widely interpreted as pattern-recognition ligands capable of engaging innate immune pathways [54,55,56]. In a skin-targeted nutrition context, this matters because barrier status and inflammatory tone are immunologically regulated, but it also requires discipline: receptor activation in vitro or in immune cells is not the same as a demonstrated skin benefit after eating a mushroom. The more defensible chain is dietary fungal polysaccharide → intestinal exposure and microbial fermentation → local immune sensing → systemic inflammatory tone → skin barrier or inflammatory endpoints.

Recent food science reviews of mushroom polysaccharides emphasize that anti-inflammatory and immunomodulatory effects depend on structural features, intestinal exposure, and host–microbiota interactions [30]. A practical consequence is that molecular weight ranges should be reported as dose–response variables. High-molecular-weight β-1,3/1,6-glucans tend to favor Dectin-1 engagement and innate immune sensing, whereas partially hydrolyzed or lower-molecular-weight fractions can shift the balance toward gut microbiota fermentation and SCFA output, with downstream consequences for skin-relevant inflammatory tone and barrier biology. Fungal polysaccharides are therefore best handled as processing-sensitive macromolecular food ingredients.

### 3.3. Cordycepin and Nucleosides: Defined Small Molecules with Limited Oral Skin Evidence

Cordycepin is a purine nucleoside, 3′-deoxyadenosine, originally isolated from *Cordyceps* cultures and now firmly associated with cultivated *Cordyceps militaris* [33]. It occurs in fruiting bodies, in vitro mycelium, and liquid fermentation products, so its upstream raw material is a continuum of biological forms while the purified end-product is a single molecule. Cultivated *C. militaris* is attractive because fruiting body culture and submerged fermentation provide controllable supply chains.

Cordycepin accumulation depends on strain genetics, medium composition, carbon/nitrogen ratio, minerals, light, and temperature, and substrate choice has been shown to alter cordycepin and ergothioneine levels in *C. militaris* fruiting bodies [11], so its content is itself a useful quality and process indicator. Mechanistically, it is linked to anti-inflammatory, antioxidant, and stress response activity. However, direct oral skin evidence is weak, most data derive from cell and animal pharmacology, oral stability, and first-pass metabolism remain concerns, and skin-specific human intake data are essentially absent. Cordycepin is therefore promising but under-validated. A recent metabolomic study of dried *Cordyceps militaris* products further illustrates that product state and consumption context can reshape metabolic profiles, flavor quality, and health-related attributes, supporting the interpretation of *Cordyceps* materials as process-defined foods [57].

Cordycepin is also a good example of why biosynthetic context matters. Genomic and transcriptomic work has begun to map cordycepin biosynthesis in *C. militaris* [33], and the discovery that fungal cordycepin biosynthesis is coupled to production of the safeguard molecule pentostatin explains how the producing fungus avoids self-toxicity from an adenosine analog [58]. These findings make cordycepin attractive for fungal biotechnology and fermentation control, but they do not solve the dietary skin evidence gap.

Mechanistic reviews support anti-inflammatory actions of cordycepin across NF-κB, MAPK, and related pathways [34], yet most evidence remains pharmacological or cell-based. This review therefore uses cordycepin mainly as a process-controllable fungal nucleoside whose skin relevance is mechanistically plausible but orally under-validated.

### 3.4. Phenolics: Low-Abundance but Processing-Sensitive Antioxidants

Phenolics are compositionally complex but comparatively low-abundance compounds in edible fungi, occurring mainly in fruiting bodies but also in mycelia and spent substrates [35]. They are dominated by phenolic acids, especially hydroxybenzoic and hydroxycinnamic acids, with occasional reports of flavonoid-like compounds whose abundance, stability, and endogenous fungal origin require case-by-case confirmation [4]. Recent review work on polyphenol classification and antioxidant assays provides a useful reminder that chemical antioxidant capacity is method-dependent and should not be equated with in vivo oral efficacy without bioaccessibility, metabolism, and endpoint validation [59].

A methodologically important point is that a substantial fraction of fungal phenolics may be bound to the cell wall matrix and released only by acid, alkaline, or sequential acid–alkali hydrolysis. Free-phenolic assays can therefore underestimate true content and partly explain inter-study variability. Phenolic profiles are species-specific, tissue-dependent, and processing-sensitive: cultivar, substrate, boiling, roasting, and drying can all change extractable phenolics [4]. For this review, their relevance is best understood as processing- and matrix-dependent; antioxidant assay activity alone does not establish in vivo relevance after oral intake.

### 3.5. Triterpenoids: Anti-Inflammatory and Matrix-Related Candidates with Exposure Limitations

Triterpenoids, especially lanostane-type triterpenes from *Ganoderma*, are structurally complex C30 compounds varying by oxidation state and substitution [38]. They have a strong mechanistic literature in inflammation, redox regulation, and extracellular matrix protection [60]. For a food-focused review, however, they should not dominate the argument, because low aqueous solubility, uncertain bioavailability, and the gap between pharmacological doses and realistic dietary exposure limit interpretation. Their evidence should be separated into ordinary food exposure, supplement exposure, formulation-enhanced exposure, and pharmacological testing.

The *Ganoderma* case has also become more chemically and biosynthetically tractable. Reviews of ganoderic acid biosynthesis and molecular mechanisms place these triterpenoids within the mevalonate–lanosterol-derived triterpene framework and summarize their links to NF-κB, MAPK, PI3K/Akt, and redox pathways [39,61]. A recent study of *Ganoderma resinaceum* isolated lanostane-type triterpenoids and showed that selected compounds could attenuate UV-induced oxidative and matrix-related injury in skin-relevant models through Nrf2 and MAPK signaling [40]. This strengthens mechanistic plausibility, but because these studies are not dietary human trials, they are treated as mechanistic support, not evidence of oral efficacy.

Work using UPLC-MS/MS and component knock-out approaches also illustrates how *Ganoderma* activity can be connected to defined chemical profiles rather than generic extract identity [62], and reviews of sesquiterpenoids and diterpenoids from *Ganoderma* species highlight the broader structural and biosynthetic richness of this genus [63]. For oral food science interpretation, however, such chemical diversity should be coupled to solubility, bioavailability, exposure realism, and safety evaluation before being translated into functional food claims.

## 4. Food Processing, Fermentation, and Biomanufacturing

The strongest food science contribution of this review lies in showing that fungal bioactives are not fixed entities. Their deliverable dose is shaped by a processing trajectory that begins with raw material formation during cultivation, continues through stabilization during drying and storage, proceeds through release during cooking or extraction, and is finally constrained by recovery, concentration, and standardization through fermentation, biomanufacturing, and quality control. This section therefore follows a food system logic: raw material formation → stabilization → release → recovery and concentration → standardized production.

### 4.1. Cultivation and Substrate Effects

Species and strain set the baseline bioactive profile [4], but substrate composition can shift polysaccharide, phenolic, ergothioneine, and nucleoside content, as shown for cordycepin and ergothioneine in *Cordyceps militaris* fruiting bodies cultivated on different substrates [11]. Fruiting bodies, submerged culture mycelia, fermentation broths, and spent substrates are distinct raw materials, even when they yield the same nominal compound. For lignocellulose-grown species, degradation and transformation of substrate-derived phenolic precursors may participate in fruiting body phenolic accumulation, illustrating that cultivation is itself a biosynthetic variable.

### 4.2. Drying, Cooking, and Storage

Thermal and post-harvest processing can preserve, degrade, transform, or release bioactives, and different compound classes respond differently. For ergothioneine, hot-air drying and pasteurization can change retention: suitable drying can preserve high levels in *Pleurotus*, whereas canning of button mushroom causes marked losses [64]. Extraction conditions modulate recovery further. For polysaccharides, processing alters extractability and molecular weight distribution; for phenolics, cooking redistributes free and bound fractions; and for cordycepin, thermal and digestive stability are limiting considerations. Because the eventual product is consumed, food-like processing should be prioritized over purely analytical extraction when judging what consumers actually ingest.

Several processing studies illustrate why this section is essential to the food science argument. Extraction solvent, cooking procedure, and storage condition can change both ergothioneine and phenolic recovery in *Flammulina velutipes* [36]. Hot-air drying and pasteurization have been reported to alter ergothioneine retention across edible mushrooms, with retention patterns differing between species and processing formats [64]. In *Pleurotus citrinopileatus*, drying mode and high hydrostatic pressure affected ergothioneine extraction, showing that the measurable dose of a fungal antioxidant is a function of both biological content and process design [65]. For phenolic-oriented ingredients, drying and extraction optimization similarly change the antioxidant profile of *Pleurotus ostreatus* [37]. The implication for skin-targeted dietary products is that ‘mushroom extract’ becomes a reproducible unit only once the processing pathway and marker recovery are specified. Table 3 compiles representative effects of cultivation, drying, cooking, extraction, and pressure processing on the major bioactive classes, underscoring that the deliverable dose of each compound is set jointly by biological content and process design. For *Cordyceps militaris*, metabolomic profiling of dried products has similarly linked consumption timing, health-related attributes, and flavor quality, reinforcing that fungal foods should be evaluated through product-state and process-defined markers [57].

### 4.3. Fermentation and Mycelial Cultivation

Fermentation and mycelial culture provide controlled, season-independent routes to fungal bioactives, often with more consistent ingredient quality than seasonal fruiting bodies. Submerged systems have been used to enrich ergothioneine in both non-fungal and fungal native producers: the bacterium *Methylobacterium aquaticum* 22A reached about 10 mg/L on methanol [66], while the basidiomycete *Panus conchatus* reached 148.79 mg/L with histidine/methionine/cysteine supplementation [67]. Submerged systems are also important to cordycepin production from *C. militaris* broth, where biosynthetic and regulatory dissection of the pathway has clarified how culture conditions influence yield [33], and exopolysaccharides can be generated in parallel. Mycelial systems are particularly attractive when a defined and repeatable polysaccharide or nucleoside profile is required for standardization. Food fermentation studies outside the fungal category also show that mixed microbial fermentation can simultaneously modify nutritional composition, flavor, and health-related effects, underscoring the need to evaluate fungal fermentation products as integrated food matrices [68].

### 4.4. Biomanufacturing and Cell Factories

Ergothioneine is the clearest example of a fungal origin bioactive moving from mushroom extraction to engineered microbial cell factories. Natural production depends on ergothioneine-rich fruiting bodies, mycelia, and processing by-products, but this route is supply-limited and content-variable; early submerged fermentation work with natural producers (Section 4.3) reached only milligram-per-liter levels. Subsequent efforts shifted to engineered hosts, including *Escherichia coli*, *Saccharomyces cerevisiae*, *Corynebacterium glutamicum,* and *Yarrowia lipolytica*. Representative reports reached gram-per-liter scale, including *E. coli* using a fungal two-enzyme pathway at 4.34 g/L [69], multi-copy-integrated *Y. lipolytica* at 7.3 g/L in a 5 L fermenter [70], and *S. cerevisiae* expressing two genes from *Grifola frondosa* at 20.61 mg/L [71]. More recent pathway reconstruction in *E. coli* has pushed production further to 7.2 g/L [72], while engineered *C. glutamicum* reached 459 mg/L under an osmotic pressure strategy [73]. Recent reviews summarize these achievements and the remaining challenges in host selection, precursor supply, purification, and regulatory acceptance [74].

The food-relevant attractions of biomanufacturing are scalable controlled fermentation, safety-compatible hosts, strain-level reproducibility, purification, and the potential use of lower-cost by-products. At the same time, high titer is not equivalent to an acceptable food ingredient: regulatory acceptance, GM-related consumer perception, impurity control, host status, and final product specification all require case-specific evaluation. Reported gram-per-liter titers indicate that engineered production is becoming technically plausible as a supply route, although food ingredient feasibility will depend on host status, purification, impurity control, production cost, regulatory acceptance, and consumer perception. If these constraints are addressed, biomanufacturing could provide a stable supply complementing natural mushroom sources.

### 4.5. By-Products and Circular Food Ingredients

Mushroom stems, processing residues, spent mushroom substrate (SMS), and fermentation side-streams are candidate sources of polysaccharides, phenolics, and other compounds, linking the topic to sustainable food systems and the circular bioeconomy. Ergothioneine in particular has been highlighted as a valorizable target in mushroom residuals [75]. Subcritical-water and pressurized-ethanol extracts of shiitake and oyster cultivation residues can retain antioxidant-active phenolics and glucan-rich fractions, and untargeted metabolomic profiling of spent oyster mushroom substrate further shows that, while ergothioneine itself is only modestly enriched in SMS relative to unused substrate, the residual matrix is differentially enriched in compounds such as cerebroside B and ganoderenic-acid-type triterpenoids that may have independent skin-relevant activity and would otherwise be discarded [76]. The appropriate framing is food ingredient valorization: SMS and stem-residue streams are entering the same regulatory and quality-control framework as conventional food ingredients, and their bioactive markers should be specified accordingly.

### 4.6. Companion-Fungus and Co-Culture Systems as an Emerging Fungal Food Paradigm

A distinctive feature of some edible fungi is that they do not grow as simple monocultures but as obligate or facultative partnerships with companion fungi. The *Tremella fuciformis*–*Annulohypoxylon stygium* system, used in commercial white jelly mushroom cultivation, is the clearest example: *Annulohypoxylon* performs oxidative degradation of lignocellulose while *Tremella* accumulates the consumed polysaccharides. Comparative transcriptomics with CRISPR/Cas9 functional analysis has begun to map carbohydrate-, amino-acid- and stress-defense pathways underlying this interaction [77]. Read cautiously, this remains a hypothesis-generating food biomanufacturing concept: oxidative-attack and product-accumulation functions can be split across organisms in a natural edible fungal system. Future fungal food ingredients could explore consortium-level design alongside fruiting bodies, submerged fermentation, and engineered single-host cell factories, although such co-culture systems still require compositional, safety, and regulatory validation before they become manufacturing routes.

## 5. Oral Bioaccessibility, Bioavailability, and Metabolism

A skin-relevant fungal bioactive must survive digestion, become bioaccessible, and, directly or indirectly, reach a biological target. This section applies the route discipline principle set out in Section 1.3 to the digestive, absorptive, and microbial steps that determine oral exposure.

### 5.1. Bioaccessibility After Digestion

The first questions are which compounds are released from the mushroom food matrix during gastrointestinal transit and how cooking, drying, particle size, and extraction change that release. Small molecules differ in expected gastrointestinal stability: ergothioneine is comparatively stable, whereas cordycepin and many phenolics are more labile or subject to transformation. Polysaccharides are largely not absorbed intact; their fate is partial structural change followed by microbial fermentation. Bound phenolics may be released only in the lower gut, shifting their site of action. This aligns with a broader food-bioactive bioaccessibility framing, in which processing effects, bioactive chemistry, bioavailability, safety risks, and mechanisms are jointly considered for food-derived bioactive ingredients [50,59].

Standardized digestion methodology is therefore essential. The INFOGEST consensus method and its updated static protocol provide widely used frameworks for simulating oral, gastric, and intestinal digestion under comparable conditions [78,79]. Despite their wide adoption in dairy, plant protein, and cereal research, INFOGEST-based studies of cooked mushrooms, mushroom powders, mushroom extracts, and fermented mycelial ingredients remain rare, and almost none have been coupled to colonic fermentation simulators or multi-omics readouts. Applying such methods to these mushroom formats would help distinguish analytical content from bioaccessible content, especially for bound phenolics, cell wall-associated ergothioneine, nucleosides, and high-molecular-weight polysaccharides. We recommend integrating INFOGEST digestion with targeted metabolomics, and adding colonic fermentation models where relevant, especially for polysaccharides and bound phenolics, as a core methodological framework for processing-aware fungal skin nutrition research.

### 5.2. Absorption and Systemic Distribution

Ergothioneine is the strongest candidate for systemic absorption and skin delivery because of dedicated OCTN1-mediated transport, high stability, and accumulation in relevant tissues [27,42]. Phenolics and cordycepin require careful treatment because absorption, conjugation, and first-pass metabolism may limit parent-compound exposure reaching skin. Polysaccharides are most plausibly indirect actors, influencing skin through gut microbiota and immune tone. Triterpenoids face solubility- and bioavailability-limited exposure at dietary doses.

### 5.3. Gut Microbial Transformation

Fungal beta-glucans and heteropolysaccharides are not expected to reach the skin intact. Their oral relevance depends on release from the food matrix, partial structural modification during digestion, access to colonic microbial fermentation, and production of short-chain fatty acids or other microbial metabolites. For *Tremella* and beta-glucan-rich materials, this places the primary exposure site in the gut rather than at the skin surface. The downstream gut–skin signaling mechanisms are discussed in Section 7.1.

### 5.4. Dose Realism

**Figure 2:**
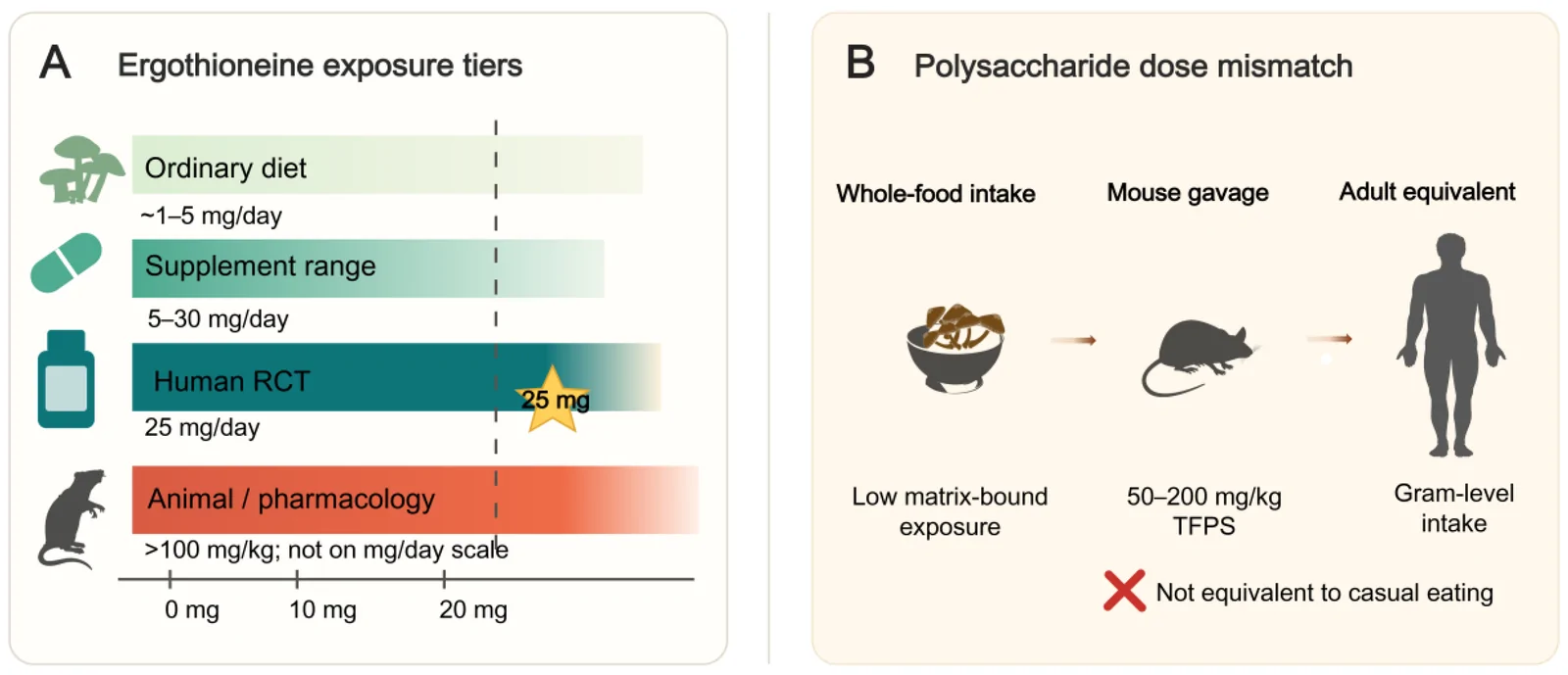
Conceptual exposure ladder for skin-targeted edible fungi bioactives. (**A**) Ergothioneine exposure differs across ordinary diets, functional food use, supplement ranges, human intervention trials, and animal/pharmacological models. (**B**) Fungal polysaccharide studies often use purified high-dose gavage models that do not map directly onto casual whole mushroom intake. The figure provides a qualitative dose category framework that complements the numerical translations in Supplementary Table S2.

Ergothioneine illustrates the problem with the firmest numbers. Estimated mean dietary intake has been reported at approximately 1.1 mg/day in the United States and 4.6 mg/day in Italy [80], whereas ergothioneine supplements and EFSA safety assessments commonly span approximately 5–30 mg/day [81]. The positive human mushroom trial delivered 25 mg of ergothioneine/day from *Pleurotus* tablets for 12 weeks [25], a dose near the top of the supplement range and several-fold above the typical dietary intake. Using reported ergothioneine contents of approximately 0.4–2.7 mg/g dry weight in *Pleurotus* and *Lentinula* materials [4], this trial dose corresponds roughly to 9–60 g of dry weight, or about 90–600 g of fresh mushrooms per day (approximately 3–7 servings), indicating that it is not achievable through casual eating alone. For polysaccharides, the gap is compounded by units: oral skin studies are largely rodent gavage studies expressed per kg of body weight; allometric conversion still often implies gram-per-day quantities of purified polysaccharide for adults. For example, the *Tremella* polysaccharide doses used in oral mouse skin models, 50–200 mg/kg/day, correspond approximately to 0.24–0.97 g/day of purified polysaccharide for a 60 kg adult after body-surface-area conversion, illustrating that animal efficacy doses cannot be equated with casual *Tremella* food intake. Supplementary Table S2 provides the numerical dose translations and approximate whole-food or human-equivalent quantities. Figure 2 presents these dose categories as a conceptual exposure ladder across ordinary foods, functional foods, supplements, and pharmacological models, emphasizing why dose category and route must be specified before skin-related claims are interpreted.

## 6. Oral Evidence Linking Edible Fungi to Skin Outcomes

Peer-reviewed human evidence linking dietary edible fungi to skin endpoints remains limited, but it is no longer restricted to a single *Pleurotus* trial. The strongest biomarker-linked mushroom material evidence comes from ergothioneine-rich *Pleurotus*, while smaller exploratory trials of *Flammulina velutipes* and *Sparassis crispa* report hydration or TEWL signals without comparable exposure biomarkers. *Tremella* polysaccharide has mechanistically informative animal oral evidence, while cordycepin, phenolics, and *Ganoderma* triterpenoids remain largely supported by topical, cell, or pharmacological studies. Table 4 summarizes the identifiable oral evidence, adjacent evidence, and explicit gaps.

### 6.1. Human Intervention Evidence

Several oral human studies have now reported skin-related endpoints for edible fungal materials, but their evidential weight differs substantially. The strongest peer-reviewed signal remains the randomized, double-blind, placebo-controlled trial of Hanayama et al. [25], in which 80 healthy women received tablets of an ergothioneine-rich *Pleurotus* strain delivering 25 mg of ergothioneine/day for 12 weeks. Stratum corneum moisture was significantly higher than the placebo at selected sites/time points; VISIA wrinkle and texture scores improved; and plasma ergothioneine rose 4.7-fold and correlated with selected moisture and TEWL parameters. These results support an oral route for an ergothioneine-rich mushroom material, but they do not establish that ordinary mushroom consumption supplies an equivalent dose.

Two smaller peer-reviewed edible fungal trials broaden the human evidence base but should be interpreted as exploratory. Nagae et al. [82] tested a *Flammulina velutipes* food matrix containing 3 g/day of dry powder for 4 weeks in a randomized, double-blind, placebo-controlled study of 30 healthy adults and reported a greater increase in skin hydration than the placebo, while TEWL did not clearly improve and neither glucosylceramide nor ergothioneine exposure was measured. Kimura et al. [29] reported a randomized, double-blind, placebo-controlled *Sparassis crispa* study (*n* = 26, 4 weeks) in which cheek TEWL was lower than the placebo, but the report was brief and no exposure biomarker was included. Both studies are valuable because they test edible fungal materials in a food form, yet they lack the dose control and biomarker linkage achieved in the *Pleurotus* trial.

A second category comprises purified or multi-ingredient ergothioneine evidence. A 2025 medRxiv preprint on DR.ERGO L-ergothioneine used 30 mg/day for 8 weeks in 66 women aged 35–59 years and reported improvements in melanin, erythema, glossiness, elasticity, wrinkles, and spots [26]. This supports ergothioneine as a candidate ingredient; however, because the material is purified/supplemental ergothioneine rather than a mushroom food, the article has not been peer-reviewed, and the trial registration descriptions are not fully consistent across sources (NCT06886061; UMIN000055792), it is treated here as provisional evidence for a fungal origin bioactive. An adjacent *Current Developments in Nutrition* abstract reported skin condition improvements after an oral formula containing ergothioneine, pyrroloquinoline quinone, and nicotinamide riboside [84], but this multi-ingredient design prevents attribution to ergothioneine or fungi. Other low-confidence human reports on mushroom-derived products were identified but were excluded from the core synthesis pending verification of source quality, trial registration, and ingredient definition.

### 6.2. Animal Oral Evidence

Animal studies provide plausibility, not consumer-relevant proof. The most pertinent polysaccharide example is Xie et al. [28], who compared oral and topical *Tremella fuciformis* polysaccharide in a DNFB-induced atopic-dermatitis-like mouse model. Oral administration at 50 or 200 mg/kg for 10 days improved TEWL, epidermal thickening, and ear edema, and was associated with regulatory T-cell, gut microbiota, and fecal metabolite changes. Additional D-galactose skin-aging mouse studies suggest that *Tremella* polysaccharides can mitigate hydroxyproline and hyaluronic acid loss, oxidative stress, inflammation, and gut metabolite disturbances, with molecular weight affecting efficacy [85,86,87]. These studies support gut–skin and systemic redox plausibility but remain preclinical.

Oral *Pleurotus* material also protected mouse skin barrier and moisture-related endpoints under UVB challenge, with ergothioneine exposure linked to plasma and epidermal delivery [83]. In contrast, no convincing oral in vivo skin study was identified for cordycepin/*Cordyceps militaris* or for *Ganoderma* triterpenoids in the literature reviewed here; their skin evidence remains mainly topical, in vitro, or pharmacological.

### 6.3. Current Evidence Position and Benchmarking

Using the evidence-grading framework defined in Section 1.5, current peer-reviewed human evidence includes one Level 1a study of ergothioneine-rich *Pleurotus* and two Level 1b exploratory trials of *Flammulina velutipes* and *Sparassis crispa* without exposure biomarkers. The DR.ERGO study is treated as provisional Level 2 human evidence because it remains a preprint and tests purified/supplemental ergothioneine, not a mushroom food. EGT-containing multi-ingredient formulas are treated as adjacent evidence, not mushroom-specific evidence.

A useful way to interpret this hierarchy is to compare edible fungi with better-established oral skin health ingredients. Collagen peptides have multiple randomized trials and meta-analyses focused on wrinkles, hydration, and dermal matrix outcomes [15,16,17]. Carotenoids such as astaxanthin have systematic reviews but mixed and endpoint-dependent evidence [18]. Broader photoaging supplement meta-analyses suggest that collagen, flavanols, and some polyphenols have stronger evidence than many other supplement classes, while several categories remain under-supported [19]. Edible fungi currently sit behind these benchmark ingredients in human evidence, but ahead in food technology distinctiveness because they combine fermentation, cell wall polysaccharides, ergothioneine production, and circular fungal biomass. Supplementary Table S3 provides the detailed benchmarking matrix for these better-established oral skin health ingredient classes.

Two benchmark contrasts are particularly relevant for this review. First, oral hyaluronan has reached a level of human testing that *Tremella* polysaccharides have not: both are associated with hydration narratives, but hyaluronan trials directly measure skin hydration, wrinkles, tone, or epidermal structure in humans [20,21], whereas *Tremella* evidence remains mainly animal, microbiota-linked, or topical. Second, carotenoid and probiotic trials illustrate route-specific endpoint design. Lycopene/lutein studies link intake to UV-responsive skin biology [22], and *L. paracasei* NCC 2461 links intake to skin sensitivity and barrier-recovery endpoints [23]. Edible fungi should be advanced with the same discipline: defined material, measurable exposure biomarker, route-relevant mechanism, and skin endpoint in the same study.

### 6.4. Risk of Bias and Quality of the Key Studies

Because Level 1a and Level 1b evidence is so limited, the conclusions of this review are disproportionately sensitive to the quality of a few pivotal studies. These studies were therefore appraised informally against route-appropriate frameworks, including Cochrane RoB 2 concepts for randomized human trials and SYRCLE-type considerations for animal studies. Supplementary Table S4 summarizes this domain-level appraisal for the key human and animal studies. The strongest study, Hanayama et al. [25], is a randomized, double-blind, placebo-controlled trial, the most robust design available here. Several features still temper its weight: a modest sample (*n* = 80), single sex (women), single mushroom strain/product, 12-week duration, multiple skin sites and endpoints assessed without a clearly described correction for multiplicity, and potential sponsor- or product-related concerns typical of commercial ingredient trials. The *Flammulina* and *Sparassis* trials broaden the edible fungal human evidence base, but their short duration, small samples, and lack of exposure biomarkers keep them in an exploratory Level 1b category. They should not be placed alongside the biomarker-linked *Pleurotus* trial. The DR.ERGO study [26] should accordingly remain provisional adjacent human evidence rather than confirmation.

The supporting animal studies [28,83,85] provide mechanistic plausibility but carry the usual preclinical caveats: small group sizes, incomplete reporting of randomization, allocation concealment and blinding, single-laboratory results without independent replication, and doses that require allometric scaling before they can be related to human intake (Section 5.4). Mechanistic cell and chemical assay studies sit lowest in the hierarchy and cannot, on their own, support oral skin claims. Two consequences follow. First, the present conclusions remain hypothesis-strengthening, particularly for polysaccharides, cordycepin, phenolics, and triterpenoids. Second, future trials should be pre-registered, use validated skin endpoints together with exposure biomarkers, report the full set of risk-of-bias elements, and include independent, non-industry replication before repeated-intake skin benefit claims are made for dietary edible fungi.

## 7. Mechanisms Under Oral Exposure and Food Matrix Constraints

Mechanistic evidence is most useful when connected to route plausibility (Section 1.3). Pathway evidence is therefore interpreted here by asking whether the compound or metabolite is bioavailable after oral intake, whether the pathway is relevant to skin endpoints, whether the supporting data come from oral studies or from topical/in vitro systems, and whether the dose is realistic.

### 7.1. Gut–Skin Axis

For fungal polysaccharides and dietary fibers, the most coherent oral mechanism is: fermentable polysaccharide → gut microbiota remodeling → short-chain fatty acid production and immune regulation → reduced systemic inflammatory tone → improved barrier and inflammatory responses. This axis is the strongest systemic rationale for *Tremella* and β-glucan-type materials and reframes their long-standing moisturizing reputation in food terms.

This model is no longer only conceptual. SCFAs produced from dietary fiber can influence epidermal metabolism and differentiation, providing a direct experimental bridge between gut fermentation and skin barrier integrity [31]. In inflammatory skin conditions, SCFAs have been discussed as immune–metabolic signals that can act through GPR43/GPR41, HDAC inhibition, and Treg-associated mechanisms [88]. For edible fungi, the most important implication is that polysaccharide structure, fermentability, and microbial metabolite output should be measured directly in skin nutrition studies. Adjacent food health evidence further supports this logic: diet composition reviews link dietary inputs to gut microbiota and associated disease mechanisms [89], food polysaccharides have been shown to affect gut bacteriome, mycobiome, and metabolome [52], soy polysaccharide can preserve colonic homeostasis through microbiota and epithelial regeneration [53], and *Bifidobacterium breve* can alleviate imiquimod-induced psoriasis in mice through secondary bile acid production and FXR-TLR4/NF-κB signaling [90]. These studies are not edible fungi skin trials, but they support the mechanistic plausibility of a gut-mediated route from food substrates to skin-relevant inflammatory and barrier outcomes.

### 7.2. Systemic Antioxidant Defense and the Nrf2/Keap1 Axis

Oxidative stress is relevant to skin aging, inflammatory amplification, and impaired cell function, but the food science interpretation must start from exposure. Ergothioneine is the strongest small-molecule candidate because it is absorbed, transported, and accumulated in tissues [41]. *Tremella* polysaccharide studies support systemic antioxidant and inflammatory remodeling in animals, whereas phenolics and *Ganoderma* triterpenoids remain mechanistically plausible but less route-validated. These pathways are therefore most useful for selecting quality markers, exposure biomarkers, and clinical endpoints; they cannot carry stand-alone efficacy claims in the absence of oral exposure data.

### 7.3. Inflammation, NF-κB, and MAPK/AP-1

NF-κB and MAPK/AP-1 signaling connect oxidative and inflammatory stress to matrix turnover and barrier-related responses, but most fungal evidence remains cellular, topical, or pharmacological. Cordycepin, *Ganoderma* triterpenoids, β-glucans, and ergothioneine can be linked to inflammatory signaling in experimental systems [38,60,91,92], yet oral interpretation requires measured intake, digestion, absorption, microbial transformation, or systemic exposure. For food ingredients, these pathways justify route-specific biomarkers; cell-based activity alone cannot establish efficacy.

### 7.4. Pigmentation, Photoaging, and Extracellular Matrix Protection

Pigmentation, photoaging, and extracellular matrix outcomes are useful skin endpoints only when they are linked to achievable dietary exposure. The canonical photoaging pathway, in which UV exposure increases reactive oxygen species, activates AP-1 and NF-κB, induces matrix metalloproteinases, and impairs collagen homeostasis, is well established outside the mushroom field and provides the mechanistic benchmark against which fungal ingredients should be judged [93,94]. Within this framework, ergothioneine has the clearest systemic route plausibility, whereas phenolics, cordycepin, and triterpenoids have pathway evidence but limited oral validation. UV-related models and *Tremella* photoaging studies provide additional biological plausibility [87,95], but they do not show that ordinary mushroom intake reverses photoaging. For edible fungi ingredients, the practical task is to connect a defined food matrix and processing route to measurable exposure biomarkers and validated endpoints such as TEWL, hydration, erythema, pigmentation balance, or matrix-related markers.

### 7.5. Barrier Function and Hydration

For oral routes, barrier and hydration outcomes are best explained through gut-mediated immune modulation, systemic redox balance, and inflammatory tone, with secondary effects on TEWL and stratum corneum hydration. The SCFA-mediated keratinocyte and immune mechanisms are discussed in Section 7.1; here, the main point is interpretive discipline. Direct film-forming and water-binding explanations belong to topical use and should not be transferred to dietary interpretation without route-relevant exposure evidence.

## 8. Route Boundaries

### 8.1. Topical Evidence as Boundary-Setting, Not Dietary Evidence

Topical applications are included here as a boundary-setting food–cosmetics interface rather than as a second evidence stream for dietary claims. Macrofungal extracts, polysaccharides, phenolics, triterpenoids, and fungal-derived formulations have been widely studied in topical anti-aging, moisturizing, pigmentation, wound-healing, and enzyme-inhibition contexts, with endpoints such as tyrosinase, elastase, hyaluronidase, collagenase, melanogenesis, oxidative stress, inflammatory signaling, fibroblast activity, keratinocyte viability, and extracellular matrix turnover [5,6,96]. These studies are scientifically useful because they identify skin-relevant pathways and material properties, but they cannot be used directly to infer dietary efficacy.

For a food-focused review, topical findings must be read under the route discipline principle set out in Section 1.3. Film formation, water binding, surface hydration, direct enzyme inhibition and local antioxidant activity after skin application therefore carry no weight for oral interpretation without route-relevant exposure evidence. This distinction is especially important for *Tremella* polysaccharides, *Ganoderma* triterpenoids and mushroom phenolics. *Tremella fuciformis* extract has shown topical formulation value, including improved hydration and reduced TEWL in emulsion testing, and *Tremella*-derived exopolysaccharides have increased moisturizing and antiwrinkle-related factors in skin cell models [97,98]. In an oral framework, however, the same material class should instead be evaluated through fermentability, microbiota-derived metabolites, immune modulation, and barrier endpoints.

A similar caution applies to *Ganoderma* and phenolic-rich mushroom materials. *Ganoderma lucidum* polysaccharide has been reported to attenuate UVB-induced melanogenesis through cAMP/PKA and ROS/MAPK-related pathways in experimental skin pigmentation models, and *Ganoderma resinaceum* triterpenoids have been linked to Nrf2 and MAPK signaling in UV-induced photoaging models [40,99]. Such findings strengthen mechanistic plausibility and help identify candidate pathways, but they do not show that ordinary dietary exposure or a realistic supplement dose reaches skin-relevant targets. Oral claims require exposure evidence, not only pathway activity in local or cell-based systems.

### 8.2. Using Topical Studies to Inform Oral Trial Design

Topical and cell-based studies should therefore be treated as hypothesis-generating and endpoint-informing evidence. They can help prioritize candidate bioactives, define plausible skin mechanisms, and select outcome measures for future oral trials. For example, topical or cell-based evidence involving oxidative stress, melanogenesis, inflammatory signaling, or matrix degradation may justify oral trial endpoints such as TEWL, stratum corneum hydration, erythema, pigmentation balance, wrinkle parameters, inflammatory biomarkers, or photoaging-related molecular markers. Formulation studies can also inform quality markers by identifying which molecular weight ranges, phenolic profiles, polysaccharide fractions, or triterpenoid groups interact most strongly with skin-relevant pathways.

The key translational point is to assign topical evidence its correct evidential role. Topical studies can guide marker selection, mechanism mapping, and endpoint design; they are not confirmatory evidence for dietary skin benefits. Oral edible fungi products should be evaluated through intake level, processing history, bioaccessibility, exposure biomarkers, gut–skin mediators, and validated skin outcomes. Maintaining this boundary prevents overclaiming while allowing the richer topical literature to guide more rigorous food-based skin nutrition research.

## 9. Safety and Regulatory Considerations

### 9.1. Oral and Food Safety Background

Because the present review concerns oral use, the relevant safety baseline is dietary and food-regulatory. Ergothioneine has a supportive food-relevant safety context, including US GRAS status and EFSA assessments for dietary supplement use [81]. Marone et al. [100] reported that a nature-identical L-ergothioneine preparation was not mutagenic in bacterial reverse-mutation assays and was well tolerated in a 90-day rat study, with a NOAEL of 1600 mg/kg bw/day. Recent reviews of edible mushroom polysaccharides [101] and of *Ganoderma lucidum* chemistry, benefits, and safety [102] report no marked acute toxicity within the tested ranges. These data support, but do not complete, an oral use case: species, processing, active content, and target population all matter. The medicine–food homology perspective is therefore useful only when traditional use is converted into modern food science evidence: resource history should generate hypotheses, whereas quality markers, route-specific exposure, dose realism, toxicology, and claim boundaries must carry the safety and functional food argument [103].

### 9.2. Contaminants, Allergenicity, and Raw Material Controls

Contaminant control deserves explicit treatment because mushrooms are efficient mineral accumulators and because skin-targeted nutrition often uses powders, extracts, or concentrated preparations well beyond casual culinary servings. Recent reviews of trace element concentrations in edible mushrooms identify cadmium, mercury, and, to a lesser extent, lead and arsenic as toxicologically important elements, especially in wild-growing species and polluted locations [104]. Species- and site-specific surveys further show that some mushrooms can accumulate high levels of cadmium and other heavy metals when grown on contaminated substrates, with cap and stipe differing in their accumulation [105]. Cultivated mushrooms are generally more controllable than wild harvests, but substrate formulation, casing material, water, geographic origin, and post-harvest processing still become part of the ingredient specification. For fungal skin nutrition products, therefore, quality control should pair bioactive markers such as ergothioneine, β-glucan, *Tremella* polysaccharide molecular weight, or cordycepin with a contaminant profile covering cadmium, mercury, lead, arsenic, and relevant process residues. In addition to toxic elements, pesticide and agricultural chemical residues should be considered in cultivated mushroom ingredients, especially when substrates, casing materials, straw, manure-derived inputs, or spent-mushroom-substrate-derived streams are used. For skin-targeted fungal powders, extracts, and concentrated ingredients, pesticide residue screening should therefore be included alongside heavy metal testing and species authentication.

Allergenicity should also be framed conservatively. Food allergy to edible mushrooms appears uncommon relative to their wide culinary use, but case reports show that ingestion of cultivated *Agaricus bisporus* can trigger severe reactions and that heat-stable low-molecular-weight allergens may be involved [106]. Fungal spores and airborne molds are better-known respiratory allergens, and cross-reactivity between environmental fungal sensitization and mushroom foods is plausible in susceptible individuals. Concentrated fungal extracts, fermented mycelial preparations, and biomanufactured ingredients add further variables, including residual proteins from the producing organism. These risks do not argue against edible fungi as food ingredients; they argue for species authentication, allergen labeling where relevant, batch-level contaminant testing, and population-aware safety studies before making repeated-intake skin claims.

### 9.3. Toxicology and Process-Related Risks

Many cell-based safety data in the existing literature were generated for topical applications, usually in keratinocyte, fibroblast, or melanocyte models. For an oral review, these cell-based topical safety data are secondary. More relevant issues include gastrointestinal tolerance, sub-chronic and chronic oral safety, allergenic proteins, fungal species authentication, heavy metals or substrate-derived contaminants, residual solvents, fermentation impurities, endotoxin-like contaminants, and batch-to-batch variability. For biomanufactured ingredients, host status, purification, residual DNA/protein, and GM-related regulatory pathways require explicit attention.

### 9.4. Regulatory Boundaries and Responsible Communication

Ordinary foods, functional foods, dietary supplements, nutricosmetics, cosmetic ingredients, and medical products are governed differently across jurisdictions. A dietary history cannot be transferred wholesale to a concentrated extract, purified bioactive, or engineered fermentation product. Responsible communication is therefore essential: edible fungi may support skin-relevant nutrition, but claims of rejuvenation, whitening, or anti-aging require strong human evidence that is not yet available for most dietary fungal materials.

## 10. Translational Challenges and Future Perspectives

### 10.1. Standardization of Fungal Materials

Standardization is constrained by species and strain variation, fruiting body versus mycelium, substrate effects, processing variation, extract-versus-whole-food differences, and marker compound selection. Progress requires treating each variable explicitly, so that heterogeneous samples are no longer collapsed into a single ‘mushroom extract’.

### 10.2. Processing-Aware Quality Markers

Class-specific markers are needed for each material. Defensible examples include ergothioneine for *Pleurotus*, *Agaricus,* and *Lentinula* materials, molecular weight and monosaccharide composition for *Tremella* polysaccharides, and cordycepin for *Cordyceps militaris*. Other candidates include β-glucan content and structure for glucan-rich materials, phenolic profiles for antioxidant-oriented products, and triterpenoid profiles for *Ganoderma*-based materials. These markers should be reported together with drying, cooking, extraction, or fermentation routes. A marker-driven approach is further supported by evidence that *Ganoderma* materials require chemistry-linked activity interpretation, polysaccharide materials require structural and mucus/barrier-relevant characterization, and *Cordyceps* products require metabolomic and product state definition [48,51,57,62].

For products positioned for repeated oral intake, these bioactive markers should be accompanied by contaminant markers. A mushroom powder or extract that is rich in ergothioneine or polysaccharide but poorly characterized for cadmium, mercury, lead, arsenic, substrate residues, or fermentation impurities is not a well-defined food ingredient [104,105].

### 10.3. Clinical Evidence Gaps

Lessons from collagen peptides, astaxanthin, and broader photoaging supplement meta-analyses point to a practical trial standard: a defined fungal matrix or purified fungal origin marker, an exposure biomarker such as plasma ergothioneine or fecal SCFA shift, validated skin endpoints, and a dose that maps back to ordinary foods, functional foods, or supplements [15,16,17,18,19]. Supplementary Table S5 lists candidate oral exposure biomarkers across the five bioactive classes covered in this review, including plasma, urinary and fecal/microbial markers, and suggested sampling windows, to support such trial designs. Without this design, edible fungi will remain scientifically interesting but commercially vulnerable to the evidence gap affecting many oral skin health products.

### 10.4. Dose-Realistic Models and Multi-Omics

Future work should explicitly bridge food intake → functional food dose → supplement dose → blood/tissue exposure → skin-biomarker response, instead of reasoning from pharmacological doses. Metabolomics can quantify fungal bioactives and human exposure. Microbiome analysis can test the gut–skin axis, and skin transcriptomics or proteomics can test pathway response. The most informative single design would pair skin transcriptomics with fecal metabolomics in the same participants and time windows, so that fungal intake is connected on one side to colonic SCFA and microbial metabolite signatures and on the other side to skin barrier, redox, and inflammatory gene programs. Multi-omics designs should therefore be built around pre-specified route hypotheses.

### 10.5. From Single Actives to Mechanism-Guided Combinations

**Figure 3:**
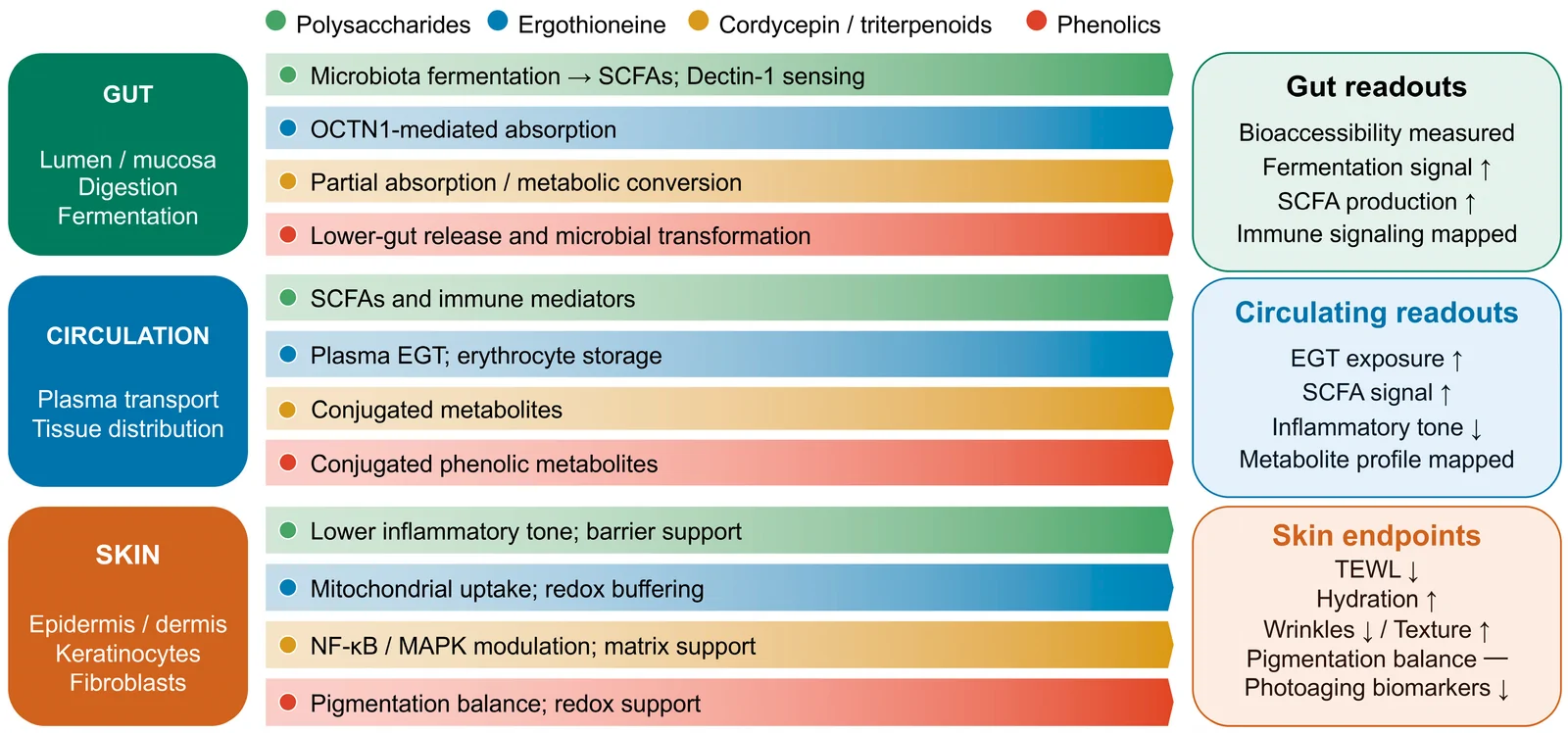
Mechanism-guided combinations of edible fungi bioactives across the gut, circulation, and skin compartments. Polysaccharides act mainly through microbiota fermentation, SCFA production, and immune–barrier regulation; ergothioneine acts through OCTN1-mediated absorption, plasma/erythrocyte storage, and mitochondrial redox buffering; cordycepin and triterpenoids act through partial absorption, metabolic conversion, and stress pathway modulation; and phenolics act through lower-gut release, microbial transformation, and conjugated metabolites. Route-specific exposure should guide mechanism-based combination design. Horizontal arrows (→) indicate directional process relationships, whereas upward and downward arrows (↑ and ↓) indicate increases and decreases in the corresponding readouts or endpoints, respectively.

The node-level differences summarized in Section 7 suggest complementary combinations: polysaccharides for gut-mediated barrier and inflammatory support, ergothioneine as a small-molecule redox buffer, cordycepin and triterpenoids for stress pathway modulation, and phenolics bridging redox and pigmentation-related processes. In an oral/functional food context, this logic should be expressed through food matrices, delivery and dose, and validated by intake-to-endpoint studies, with oral delivery kept separate from topical formulation logic. Figure 3 maps these compartment-specific mechanisms across the gut, circulation, and skin compartments, and illustrates how route discipline, not topical formulation logic, should guide combination design.

## 11. Conclusions

Edible fungi have long been associated with dietary nourishment, vitality, and skin-related wellness, but modern food science must move beyond cultural narratives and extract-based claims. A redox ecology lens provides a distinctive biological starting point for wood-associated edible basidiomycetes, while high-ergothioneine ectomycorrhizal *Boletus* and low-ergothioneine *Tremella* show that this lens is bounded. Across the five representative classes reviewed here, ergothioneine currently has the strongest oral case; fungal polysaccharides are best interpreted as gut–skin axis modulators; and cordycepin, phenolics, and triterpenoids remain mechanistically plausible but orally under-validated.

The decisive limitations lie not in mechanistic plausibility but in dose realism, oral bioavailability, processing-aware standardization, and human evidence. The field should therefore move beyond traditional skin health narratives toward standardized, processing-aware, and route-validated fungal food systems for skin-targeted nutrition.

## Figures and Tables

**Table 1:** Evidence-grading framework applied in this review.

Level	Label	Minimum Criteria
1a	Biomarker-linked human RCT	Randomized, controlled human trial with a defined fungal ingredient or purified fungal origin bioactive, validated skin endpoint(s), and a measured oral exposure biomarker linking intake to a systemic or tissue-level readout.
1b	Exploratory human RCT	Randomized, controlled human trial with a defined fungal material and skin endpoint(s), but limited sample size, short duration, or absent exposure biomarker; the results are hypothesis-generating rather than confirmatory.
2	Provisional/adjacent human evidence	Human data from preprints, purified non-mushroom ingredients, multi-ingredient formulas, or studies with weaker controls; not direct evidence for dietary edible fungi efficacy without independent peer-reviewed replication.
3	Animal oral studies	In vivo oral studies in animals with skin-relevant endpoints; useful for mechanistic plausibility and route support, but requiring dose translation before human interpretation.
4	In vitro/mechanistic studies	In vitro digestion, microbiota fermentation, cell culture, or organ models; useful for pathway identification and biomarker selection but insufficient alone to support oral claims.
5	Chemical assays/topical-only evidence	Antioxidant capacity assays, enzyme inhibition assays, topical skin cell studies, or cosmetic formulation tests; useful for hypothesis generation but not for inferring dietary efficacy.

**Table 2:** Comparative matrix of representative edible fungal bioactives for skin-targeted dietary nutrition.

Bioactive Class	Main Fungal Sources	Food Technology Variables	Most Plausible Oral Route	Skin-Relevant Targets	Evidence Level	Key Refs
Ergothioneine	*Pleurotus*, *Agaricus*, *Lentinula*; also produced by fungi/microbes beyond wood-decay species	Species, cap/stem distribution, drying, heat treatment, extraction, fermentation, cell factory production	Absorption and OCTN1-mediated distribution; systemic redox buffering	Oxidative stress, photoaging, erythema/pigmentation, barrier endpoints	Level 1a (*Pleurotus*); Level 2 provisional for purified EGT; clearest oral route	[4,25,26,27]
Polysaccharides and β-glucans	*Tremella*, *Lentinula*, *Auricularia*, *Pleurotus*, *Ganoderma*, *Sparassis*; fruiting bodies, mycelia, exopolysaccharides	Molecular weight, branching, charge, extraction route, fermentation, drying, food matrix	Microbiota fermentation, SCFAs, immune regulation, barrier-related systemic effects	TEWL, barrier dysfunction, inflammatory tone, hydration-associated outcomes	Level 1b small exploratory for *Sparassis*; mainly Level 3; no biomarker-linked human data	[13,28,29,30,31,32]
Cordycepin and nucleosides	*Cordyceps militaris* fruiting bodies, mycelia, fermentation broth	Strain, medium composition, light, temperature, fermentation, extraction	Potential absorption but metabolism and deamination are major uncertainties	Inflammatory signaling, oxidative stress, matrix-related pathways	Level 4–5; oral skin evidence sparse	[11,33,34]
Phenolics	*Agaricus*, *Pleurotus*, *Lentinula*, *Hericium*; cultivation residues/spent substrates	Free vs. bound fractions, substrate, cooking, roasting, boiling, drying, extraction	Partial release and metabolism; possible lower-gut release of bound phenolics	Antioxidant capacity, tyrosinase-related processes, pigmentation balance	Level 4–5; chemical/in vitro dominate	[4,35,36,37]
Triterpenoids	*Ganoderma* and related food-compatible medicinal fungi	Species, extraction solvent, formulation, solubility, dietary/supplement form	Limited by solubility and bioavailability; supplement exposure more realistic than ordinary intake	NF-κB/MAPK, Nrf2/HO-1, MMPs, extracellular matrix protection	Level 4 mechanistic; oral skin validation weak	[38,39,40]

**Table 3:** Effects of cultivation, processing, extraction, and biomanufacturing on the major skin-relevant fungal bioactive classes.

Bioactive Class	Processing/Production Step	Representative Matrix	Reported Effect on Content or Recovery (Indicative)	Key Refs
Ergothioneine	Hot-air drying (mild conditions)	*Pleurotus* spp., shiitake	Largely preserved; suitable drying retains high content, in contrast to harsher formats	[64,65]
Ergothioneine	Canning/pasteurization	*Agaricus bisporus*	Marked losses, partly via leaching of ergothioneine into the canning liquid	[64]
Ergothioneine and phenolics	Boiling, extraction solvent choice, storage	*Flammulina velutipes*	Recovery strongly solvent- and method-dependent; boiling leaches ergothioneine into the broth	[36]
Ergothioneine	Drying mode + high hydrostatic pressure	*Pleurotus citrinopileatus*	Pressure-assisted extraction increases measurable yield relative to conventional extraction	[65]
Polysaccharides and β-glucans	Hot-water vs. alkali extraction; thermal processing	*Tremella*, *Lentinula*, *Pleurotus*	Alters molecular weight, yield, branching, and charge, changing fermentability and immune activity, not just total content	[13,30]
Phenolics	Drying, roasting, boiling, extraction optimization	*Pleurotus ostreatus*, *Agaricus*	Redistribution of free and bound fractions; optimized drying raises extractable phenolics and antioxidant capacity	[4,37]
Cordycepin	Substrate and cultivation conditions; fermentation control	*Cordyceps militaris*	Content varies several-fold with substrate and strain; thermal and digestive stability are limiting for the oral route	[11,33]
Triterpenoids	Extraction solvent and formulation	*Ganoderma* spp.	Low aqueous solubility limits recovery; strongly solvent- and formulation-dependent, with modest dietary exposure	[38,39]

**Table 4:** Oral dietary/supplemental evidence linking edible fungi or fungal origin bioactives to skin outcomes.

Study/Material	Model and Design	Dose and Duration	Subjects	Skin Endpoints	Main Findings	Level (Section 1.5)
Hanayama et al. [25]; EGT-rich *Pleurotus* tablets	Human RCT, double-blind, placebo-controlled, parallel	25 mg EGT/day; 12 weeks	80 healthy women	Moisture, TEWL, VISIA wrinkle/texture/pore/spot scores	Moisture improved at selected sites/time points; VISIA wrinkle and texture scores improved; plasma EGT rose and correlated with some barrier/moisture parameters	Level 1a
Nagae et al. [82]; *Flammulina velutipes* dry powder	Human RCT, double-blind, placebo-controlled	3 g dry powder/day in food matrix; 4 weeks	30 healthy adults	Skin hydration, TEWL, subjective skin condition	Hydration increased more than the placebo; TEWL not clearly improved; GlcCer/EGT exposure not measured	Level 1b exploratory human RCT
Kimura et al. [29]; *Sparassis crispa*	Human RCT, double-blind, placebo-controlled; brief report with animal support	Oral *S. crispa*; 4 weeks	26 healthy subjects	Cheek TEWL	Cheek TEWL was lower than the placebo after 4 weeks; brief report and no exposure biomarker	Level 1b exploratory human RCT
Ma et al. [26]; DR.ERGO L-ergothioneine preprint	Human RCT described in preprint; registry descriptions require caution	30 mg EGT/day; 8 weeks	66 women aged 35–59 y	Melanin, erythema, glossiness, elasticity, wrinkles, spots	Preprint reports improvements in multiple skin parameters; purified/supplemental ingredient, not whole mushroom; registry descriptions not fully consistent	Level 2 provisional (preprint)
Hanayama et al. [83]; *Pleurotus* material/extract in UVB-challenged mice	Mouse oral study with UVB challenge	Dietary mushroom material; exposure verified by plasma/epidermal EGT	Mice	Moisture, TEWL, oxidative stress markers, epidermal thickness	Alleviated UVB-induced barrier dysfunction and oxidative stress; supports route plausibility via OCTN1-mediated EGT delivery	Level 3
Xie et al. [28]; *Tremella* polysaccharides	Mouse DNFB-induced AD-like model; oral vs. topical	50 or 200 mg/kg TFPS; 10 days post-DNFB	BALB/c mice	TEWL, epidermal thickening, ear edema, immune and gut microbiota endpoints	Both routes improved AD-like symptoms; oral route was more effective and associated with Treg, microbiota, and fecal metabolite changes	Level 3
*Cordyceps militaris*/cordycepin	No oral in vivo skin study identified in the core literature reviewed here	-	-	-	Skin evidence is mainly topical, cellular, or pharmacological; oral skin-targeted validation is a gap	Gap
*Ganoderma* triterpenoids and mushroom phenolics	No dietary-route skin endpoint study identified in the core literature reviewed here	-	-	-	Mechanistic and topical evidence is relevant but insufficient for dietary route claims	Gap

## Data Availability

No new data were created or analyzed in this study. All information synthesized in this review is derived from the published literature cited in the reference list and in the Supplementary Materials.

## Supplementary Materials

The following supporting information can be downloaded at: https://www.mdpi.com/article/10.3390/antiox15091111/s1, Table S1: Structured summary of literature identification, screening and study selection; Table S2: Dose-realism translation for representative skin-targeted edible-fungi bioactives; Table S3: Benchmarking edible fungi against better-established oral skin-health ingredient classes; Table S4: Risk-of-bias and quality appraisal of the pivotal oral studies; Table S5: Candidate oral-exposure biomarkers for future skin-targeted edible-fungi trials.
