# Supplementary material for: Edible Fungi and Skin Redox Homeostasis: Bioactive Diversity, Processing, Oral Exposure, and Evidence Gaps
Source: Antioxidants (Basel). 2026 Sep 2;15(9):1111. doi: 10.3390/antiox15091111 (PMC13603789; doi:10.3390/antiox15091111)
Supplement: Supplementary file 1 [file antioxidants-15-01111-s001.zip › antioxidants-4504187-supplementary.pdf]

Edible Fungi and Skin Redox Homeostasis: Bioactive Diversity, Processing, Oral Exposure, and Evidence Gaps

Caizhen Wang[1], †, Ying Wang[1], †, Hongyu Chen[1], Bing Li[1], Youran Shao[1], Gen Zou[1], *

[1] National Engineering Research Center of Edible Fungi, Key Laboratory of Applied Mycological Resources and Utilization, Ministry of Agriculture, Institute of Edible Fungi, Shanghai Academy of Agricultural Sciences, Shanghai 201403, China

† These authors contributed equally to this work.

* Correspondence: zougen@sibs.ac.cn (G. Zou)

Supplementary Materials

Note: Evidence levels cited in these tables follow the five-level hierarchy defined in Section 1.5 (Table 1) of the main manuscript.

Supplementary Table S1. Structured summary of literature identification, screening, and study selection.

Stage	Process	Outcome
Identification	Literature was identified through structured searches of Web of Science Core Collection, Scopus, and PubMed, covering publications from January 2000 to 29 June 2026. Search terms combined edible fungi or representative taxa with bioactive classes, oral exposure, skin-related endpoints, processing, fermentation, biomanufacturing, and food safety terms. Additional sources were identified through backward citation searching, reference lists of relevant reviews and primary studies, selected preprints, and targeted searches for dose realism and safety evidence. Searches focused on English-language records, while selected non-English sources, mainly Chinese-language studies, were retained when they provided uniquely relevant evidence on edible fungal processing, biomanufacturing, safety, or mechanisms.	Candidate records were assembled for title/abstract and full-text evaluation. During peer review, one review published on 29 June 2026 (the original search cutoff date) was added to contextualize the scope and novelty of the synthesis.
Screening	Titles and abstracts were screened against predefined eligibility criteria focusing on edible or food-compatible fungi, fungal origin bioactives, food processing, oral bioaccessibility/bioavailability, gut–skin mechanisms, skin-related endpoints, and food safety relevance.	Records clearly unrelated to edible fungi, oral exposure, skin-relevant outcomes, food processing, or food safety considerations were excluded.
Eligibility	Full texts of potentially relevant records were assessed for inclusion. Eligible records included human intervention studies, animal oral studies, in vitro digestion and microbiota studies, mechanistic studies relevant to oral-route interpretation, food technology studies, and analytical or safety studies relevant to standardization.	Records were excluded when they were topical-only without oral or mechanistic relevance, concerned non-edible or strictly medicinal fungi without food relevance, lacked relevant evidence or analytical value, or could not be retrieved.
Inclusion	Sources were retained when they contributed directly to at least one analytical dimension of the review: bioactive formation and composition, processing effects, fermentation or biomanufacturing, oral exposure, gut–skin mechanisms, skin outcomes, dose realism, safety, or translational standards.	The retained corpus, listed in full in the reference list of the main manuscript, comprises core primary studies together with methodological, background, mechanistic, and route-boundary sources spanning bioactive composition, food processing and fermentation, oral exposure, gut barrier mechanisms, safety, and translational standardization.

Note. This table summarizes the structured, transparent search and selection process used for a critical narrative review and is not a quantitative PRISMA flow diagram.

Supplementary Table S2. Dose realism translation for representative skin-targeted edible fungi bioactives.

Exposure scenario	Reported dose	Whole-food/human-equivalent translation	Feasible from ordinary diet?	Key refs
Ergothioneine—mean dietary intake (United States)	≈1.1 mg/day	Reference baseline for habitual intake	Baseline (achieved)	[80]
Ergothioneine—mean dietary intake (Italy)	≈4.6 mg/day	Higher habitual intake than the US, still below trial doses	Baseline (achieved)	[80]
Ergothioneine—supplement/EFSA safety range	≈5–30 mg/day	Delivered as purified or concentrated ergothioneine	Yes, via supplements	[81]
Ergothioneine—positive human trial (*Pleurotus* tablets)	25 mg/day × 12 weeks	≈9–60 g dry weight (≈90–600 g fresh, ≈3–7 servings) of ergothioneine-rich mushroom per day, based on 0.4–2.7 mg/g of dry weight	No, not achievable from casual diet	[4,25]
Ergothioneine—DR.ERGO trial (purified)	30 mg/day × 8 weeks	Purified/supplemental ergothioneine product, not a mushroom food	Supplement only	[26]
Tremella polysaccharide—mouse oral effective dose	50–200 mg/kg/day	Human-equivalent ≈4–16 mg/kg ≈0.24–0.97 g/day purified polysaccharide (60 kg adult)	Far above casual *Tremella* intake	[28]

Note. Human-equivalent doses were estimated using standard body-surface-area allometric scaling (HED = mouse dose × 3/37; mouse-to-human divisor ≈12.3). Mushroom-equivalent ranges depend on species ergothioneine content and should be treated as approximate.

Supplementary Table S3. Benchmarking edible fungi against better-established oral skin health ingredient classes.

Comparator class	Evidence maturity	Typical endpoints	What edible fungi can learn	Key refs
Collagen peptides	Relatively mature; multiple RCTs and meta-analyses	Wrinkles, elasticity, dermal matrix, hydration	Use defined ingredient doses and validated trial endpoints instead of broad beauty claims	[15,17]
Oral hyaluronic acid/hyaluronan	Moderate; multiple RCTs	Hydration, wrinkles, skin tone, epidermal thickness	*Tremella* polysaccharides need comparable oral human trials, not reliance on topical moisturizing logic	[20,21]
Carotenoids/astaxanthin	Moderate but mixed; endpoint-dependent	Moisture, elasticity, wrinkles, photoprotection	Avoid overclaiming when evidence is heterogeneous	[18]
Lycopene/lutein carotenoids	Moderate; human photoprotection evidence	UV response, erythema-related biology, photoaging gene expression	Fungal antioxidants need UV/photoaging endpoints beyond chemical antioxidant assays	[22]
Polyphenols/flavanols	Better represented in photoaging meta-analyses	MED, elasticity, oxidative stress-related endpoints	Connect bioavailability and clinical endpoints, not only antioxidant assays	[19]
Probiotics/prebiotics/SCFAs	Mechanistically strong but product-specific	Inflammation, barrier, AD-related endpoints	Test fungal polysaccharides with gut–skin biomarkers and barrier-recovery endpoints	[23,31]
Edible fungi	Narrow human evidence; strong processing/biotech rationale	Hydration, TEWL, VISIA, inflammatory/barrier models	Build dose-realistic trials using standardized fungal markers and route-specific exposure evidence	[25,28,29,82]

Supplementary Table S4. Risk-of-bias and quality appraisal of the pivotal oral studies.

Study (design; framework)	Sequence generation and allocation	Blinding	Outcome data and measurement	Selective reporting/multiplicity	Other concerns	Overall
Hanayama et al. [25]; human RCT; RoB 2	L	L (double-blind)	L (instrumental measures)	S (many sites/endpoints; multiplicity not addressed)	S (single sex, single product/strain; potential sponsor- or product-related concern if applicable)	S
Ma et al. [26]; human RCT (preprint); RoB 2	U/S (preprint and registry descriptions not fully consistent)	L (reported double-blind)	S	S (not peer-reviewed; multiple endpoints)	H (preprint; purified ingredient, not a mushroom food; registry interpretation requires caution)	S-H
Nagae et al. [82]; human RCT; RoB 2	L (block randomization)	L (double-blind)	S (small sample; hydration primary)	S (closed testing; TEWL not clearly improved)	S (single variety/food matrix; no GlcCer or EGT exposure marker; 4 weeks)	S
Kimura et al. [29]; human RCT; RoB 2	U (limited detail)	U/S (reported double-blind)	S (brief report; TEWL endpoint)	U	H (small sample; no exposure marker; limited reporting)	S-H
Xie et al. [28]; mouse oral; SYRCLE	U (randomization not described)	U (blinding not reported)	S	U	H (single laboratory; per kg dose needs allometric scaling)	U-H
Hanayama et al. [83]; mouse oral (UVB); SYRCLE	U	U	L (objective EGT/biochemical readouts)	U	S (single laboratory; potential product-related concern if applicable)	U
Xu et al. [85]; mouse oral (D-galactose); SYRCLE	U	U	S (proteomic endpoints)	U	H (single laboratory; scaling required)	U-H

Note. Studies were assessed against Cochrane RoB 2 domains (randomized human trials) and SYRCLE-type domains (animal studies). L = low concern; S = some concern; H = high concern; and U = unclear or insufficient reporting.

Supplementary Table S5. Candidate oral exposure biomarkers for future skin-targeted edible fungi trials.

Bioactive class	Plasma marker	Urinary marker	Fecal/microbial marker	Sampling window	Key refs
Ergothioneine	Plasma EGT (LC-MS/MS); erythrocyte EGT for longer-term exposure	Limited; renal reabsorption via OCTN1 reduces value	Not informative; absorbed proximally	Baseline, wk 4, wk 8/12 (steady-state)	[25,27,42]
Polysaccharides/β-glucans	Circulating SCFAs; LPS-binding protein	Tryptophan/indole derivatives where relevant	Fecal SCFAs; 16S shifts in fiber-degrading taxa	Baseline, wk 2–4, end of intervention	[28,31]
Cordycepin/nucleosides	Parent cordycepin (short half-life); 3′-deoxyinosine	3′-deoxyinosine and conjugates	Limited utility	Acute (0–6 h) + chronic baseline/endpoint	[33,34]
Phenolics	Conjugated hydroxybenzoic/hydroxycinnamic acid metabolites	Total polyphenol-derived metabolites; hippuric/protocatechuic acid	Lower-gut release of bound phenolics	24 h urine post-intake; chronic endpoint	[4,35]
Triterpenoids	Free and conjugated ganoderic acid species; oxidized lanostane derivatives	Conjugate excretion (limited)	Bile acid co-profiling for gut–liver context	Acute PK + chronic exposure	[38,39]
